# Radiation Dose Metrics and Local Diagnostic Reference Levels in Low-Dose Stent-Assisted Coiling of Intracranial Aneurysms

**DOI:** 10.3390/jcm15052059

**Published:** 2026-03-08

**Authors:** Mariusz Stanisław Sowa, Joanna Sowa, Kamil Adam Węglarz, Maciej Budzanowski

**Affiliations:** 1 Department of Neurology and Neurosurgery, Faculty of Medicine, Collegium Medicum, University of Warmia and Mazury in Olsztyn, Aleja Warszawska 30, 10-082 Olsztyn, Poland; neurochirurgia@uwm.edu.pl (M.S.S.); lekkamiladamweglarz@gmail.com (K.A.W.); 2 Independent Researcher, Aleja Warszawska 30, 10-082 Olsztyn, Poland; 3 Department of Radiation Physics and Dosimetry, Polish Academy of Sciences, 31-342 Kraków, Poland; maciej.budzanowski@wp.pl

**Keywords:** DRL, diagnostic reference levels, aneurysm treatment, stent-assisted embolization, radiation dose optimization

## Abstract

**Background/Objectives:** Operator experience, the implementation of low frame rates during both fluoroscopy and digital subtraction angiography (DSA), and the use of modern angiographic systems are essential for maintaining diagnostic image quality while minimizing ionizing radiation exposure during stent-assisted endovascular treatment of intracranial aneurysms. At the study center, a low-dose protocol is employed, using the lowest available fluoroscopy frame rate (3.125 frames per second) and a nominal acquisition rate of 2 frames per second for DSA, three-dimensional (3D) rotational angiography, 2D/3D mapping, and roadmapping. **Methods:** A retrospective analysis was performed on 132 stent-assisted procedures conducted at a single tertiary center between 2018 and 2024. For each procedure, data were collected for dose-area product (DAP), reference air kerma (Ka,r), fluoroscopy time (FT), and the total number of DSA frames. Local diagnostic reference levels (DRLs; 75th percentile [P75]) and typical values (50th percentile [P50]) were established and compared with values reported in the literature. **Results:** For all patients the P75 values, representing DRLs, were 19.89 Gy·cm^2^ for DAP, 332 mGy for Ka,r, 25 min 32 s for FT, and 354 DSA frames. The P50 values were 13.71 Gy·cm^2^ for DAP, 219.5 mGy for Ka,r, 20 min 36 s for FT, and 277 DSA frames. **Conclusions:** In this single-center cohort, dose metrics for stent-assisted coil embolization were within the lower range of published values. Cross-study comparisons remain descriptive and require cautious interpretation. The proposed local DRLs may support quality assurance, dose optimization, and patient safety in similar clinical settings. Further multicenter and multi-operator studies are necessary to assess transferability and applicability beyond coil-only procedures. Limitations include the retrospective single-center design (single operator) and the lack of a contemporaneous control group and formal image-quality/outcome assessment.

## 1. Introduction

Endovascular management of intracranial aneurysms using stent-based techniques, including both stent-assisted coiling (stent with adjunctive coils) and approaches without coil placement (single or multiple stent implantation), constitutes a cornerstone of contemporary neurointerventional therapy. Although these procedures are clinically effective, they may result in significant exposure to ionizing radiation due to extended fluoroscopy, multiple digital subtraction angiography (DSA) acquisitions, and additional imaging steps required for accurate device deployment and assessment of treatment outcomes [1,2].

In this context, dose optimization in accordance with the as low as reasonably achievable (ALARA) principle [3] and systematic monitoring of angiography-reported dose indicators, including dose-area product (DAP), reference air kerma (Ka,r), and fluoroscopy time (FT), are of particular importance. Diagnostic reference levels (DRLs) serve as practical benchmarks to identify procedures associated with unusually high doses and to promote harmonization of practice across operators and centers [4,5]. However, DRLs for neurointerventional procedures remain limited, and direct comparisons are complicated by variations in case complexity and treatment strategies, such as stent-assisted coiling, stent-only approaches, coil-only embolization, flow-diverter implantation, or woven endovascular bridge (WEB) devices [6,7].

This study aimed to characterize the distribution of radiation dose metrics in stent-based intracranial aneurysm treatment (stent-only or stent-assisted coiling) and to establish local DRLs for this specific group of interventions. Accordingly, only patients treated with stent-assisted endovascular embolization were included. A retrospective evaluation was conducted on a broad spectrum of endovascular neurointerventions performed between 2018 and 2024 at a single university medical center. The analysis considered patient sex, body weight, and age, and the results were compared with data reported in the scientific literature published between 2013 and 2025.

## 2. Materials and Methods

Retrospective data from 2018 to 2024 were utilized, and in accordance with applicable regulations, approval by the Bioethics Committee Faculty of Medicine, Collegium Medicum, University of Warmia and Mazury was not required. The cohort consisted of 132 consecutive patients treated at the University Hospital in Olsztyn for intracranial aneurysms using stent-based endovascular techniques, either with or without adjunctive coiling. The study population comprised 105 women and 27 men aged 24 to 81 years, with a mean age of 54 years (Table 1).

Table 2 presents the treatment modalities, indicating whether treatment involved only a stent, a stent with adjunctive coil embolization, or if more than one stent was deployed during the procedure.

Figure 1, Figure 2, Figure 3 and Figure 4 show Patient X, who was treated with stent-only endovascular therapy. Figure 4, Figure 5, Figure 6, Figure 7 and Figure 8 show Patient Y, who received stent-assisted coiling (stent placement with adjunctive coils).

### 2.1. Angiography Procedure Room

All interventions were performed in a dedicated angiography suite equipped with a Philips Azurion ClarityIQ b-plane w7 b20 system (Philips Medical Systems, Best, The Netherlands) featuring a 30 × 30 cm flat-panel detector, three-dimensional (3D) imaging, and advanced visualization functions. Modern angiography platforms typically provide a single, large-format monitor that supports multiple layout options, including a wide-screen configuration [6] and a conventional view that replicates the classic multi-monitor arrangement. In single-plane procedures, the wide-screen layout can reduce the need for image magnification, providing an effective method to lower radiation exposure for both patients and the clinical staff (Figure 9). The Philips Azurion system also incorporates automatic beam filtration, which continuously and dynamically introduces copper–aluminum prefilters into the X-ray beam based on patient thickness, field size, source-to-image distance, and the selected exposure settings. This filtration removes the low-energy portion of the spectrum while preserving angiographic contrast and image quality, operating in real time alongside automatic adjustment of the mean realized tube voltage (kV), tube current-time product per frame (mA), and frame rate.

### 2.2. Low-Dose Technique

All endovascular interventions were performed using an approach that prioritized the maximum feasible reduction in frame rate in both fluoroscopy and DSA, supported by modern angiographic equipment. Sustained clinical experience with low acquisition settings was also essential. The operator developed this competence through extended observation and hands-on training under a specialist who routinely performed procedures at reduced frame rates [8]. During the initial five years of a 12-year clinical practice period, the operator worked with an angiography system of lower performance than the currently used Azurion platform (installed and in routine operation since 2018). This experience increased tolerance to the less smooth image sequences inherent to low frame rates, which reflects reduced temporal resolution rather than diminished image quality, and strengthened the spatial perception required for accurate visualization of vascular pathology. As described in the “Angiographic system and quality control” section, regular quality control (QC) testing confirms that image quality under these low-dose settings remains within diagnostic requirements for neuroangiographic imaging.

**Figure 1:**
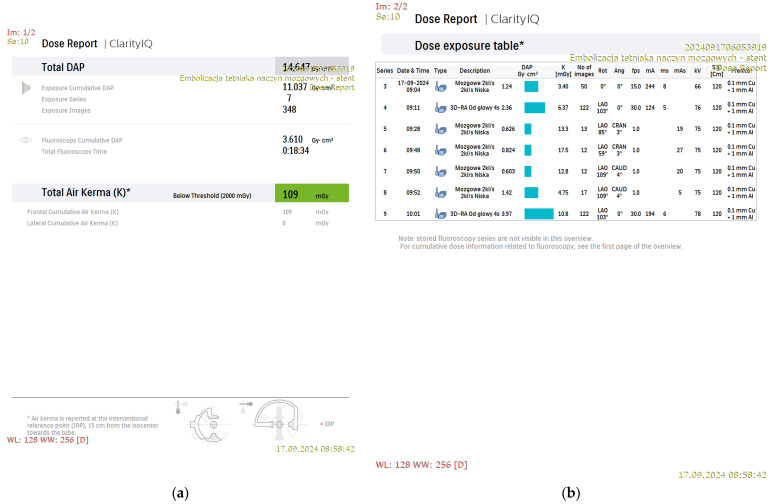
An example dose report from the stent-only treatment of an intracranial aneurysm using a single-plane system (Patient X). The first page of the system-generated dose report (**a**) and a dose exposure table (**b**) “mózgowe 2 kl/s, 2 kl/s niska” (“Cerebral 2 fps, 2 fps Low”), “3D-RA Od głowy” (“3D-RA head”), “embolizacja tetniaka naczyn mozgowych- stent” (“Endovascular embolization of a cerebral aneurysm- stent”).

**Figure 5:**
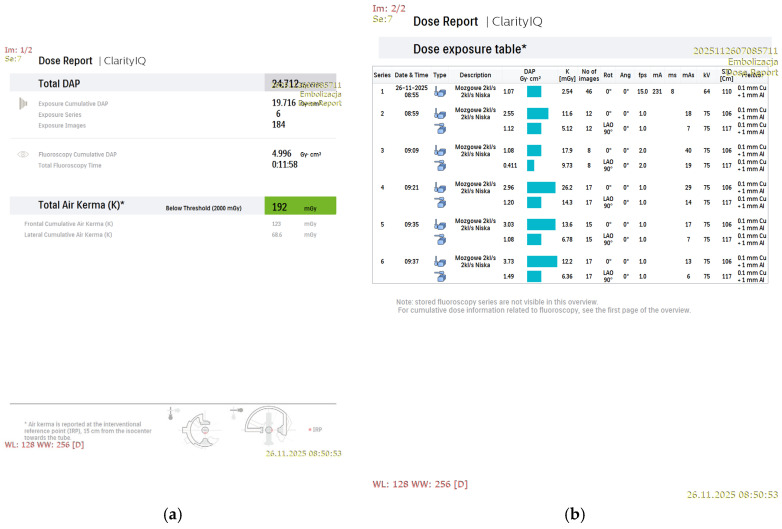
An example dose report from stent-assisted coil treatment of an intracranial aneurysm using a b-plane system in Patient Y. The first page of the system-generated dose report (**a**) and a dose exposure table (**b**) “mózgowe 2 kl/s, 2 kl/s niska” (“Cerebral 2 fps, 2 fps Low”), “3D-RA Od głowy” (“3D-RA head”), “embolizacja” (“Endovascular embolization”).

**Figure 9:**
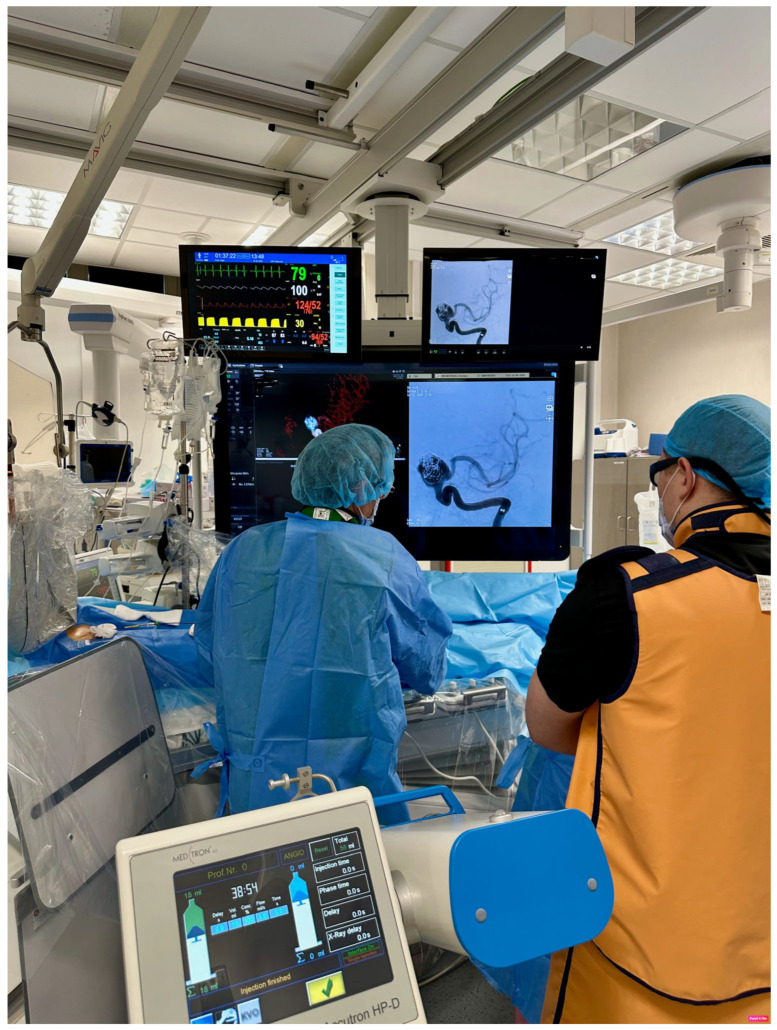
Philips Azurion biplane angiography system (W7/B20) and wide display monitor during aneurysm embolization.

The system was configured with baseline nominal frame rates of 2 frames per second (fps) for DSA and 3.125 fps for fluoroscopy to achieve the lowest feasible radiation exposure during the procedure (Figure 1b and Figure 5b). Additionally, DSA was performed using a multiphase acquisition protocol comprising three phases: an initial phase of 4 s at 2 fps, a second phase of 8 s at 1 fps, and a venous phase at 0.5 fps, further reducing patient radiation exposure.

The nominal DSA frame rates were influenced by automatic exposure adaptation (LOW mode), which was activated by the operator. The acquisition reports “Description” field includes the Polish entry “mózgowe 2 kl/s, 2 kl/s niska” (“Cerebral 2 fps, 2 fps Low”), confirming selection of LOW mode. In this mode, Azurion algorithms use inputs such as patient tissue thickness, geometric configuration (projection angles), filtration, and other parameters to automatically adjust the frame rate and exposure settings, maintaining adequate image quality while minimizing radiation dose. As a result, radiation exposure is reduced for both patients and medical staff.

The dynamics of low-dose protocol optimization, as shown in Figure 5b, demonstrate variability in acquisition frame rate across consecutive series during the procedure. In the first series, at the onset of the therapeutic intervention, the acquisition began at 15 fps despite the low-dose settings. In the second series, the frame rate was reduced to 1 fps, with a brief increase to 2 fps recorded in the third series. From series 4 to 6, the frame rate was maintained at 1 fps throughout the procedure. These fluctuations reflect algorithm-driven adaptation to changing imaging conditions.

To characterize the exposure settings achieved in clinical practice, we retrospectively extracted technical parameters from the system dose reports for all procedures included in the study.

For the DSA series, we calculated the mean realized tube voltage (kV), mAs, filtration (Sr), and the effective frame rate. The corresponding values for the whole cohort were:

- mA: [26.18 mA ± 13.8/1–65]
- kV: [74.58 kV ± 2.83/61–84]
- Sr: [0.1 mm Cu + 1 mm Al, for almost all cases]
- fps: [2.81 ± 4.55/0.5–15].

For fluoroscopy, in addition to the total FT, we used the dose report to extract the associated DAP values for all procedures:

- FT in seconds: [1389.17 ± 607.73/609–4342]
- fluoroscopy-related DAP: [6.682 ± 4.620/1.32–29.569]

The data indicate that the low-dose approach translated beyond nominal protocol settings into the exposure parameters applied in routine clinical work.

The radiation exposure per case was influenced by the imaging configuration required to adequately visualize the target lesion. Single-plane acquisition was employed when technically and clinically sufficient, whereas biplane imaging was selected for anatomies and lesion characteristics that were more complex, such as challenging aneurysm locations, significant vessel tortuosity, or intricate morphology.

### 2.3. Angiographic System and Quality Control

A structured QC program is implemented in the angiography suite in accordance with the manufacturer’s recommendations and national QC guidelines. Additionally, specialist tests mandated by the national regulatory framework are conducted on a monthly and annual basis. The system undergoes an annual service inspection to verify the technical condition of the equipment and ensure that the quality of acquired images remains within required specifications.

### 2.4. Three-Dimensional Rotational Angiography

On the Azurion platform, 3D rotational angiography (3D-RA) is performed using a semi-automated workflow. Once the operator initiates the sequence, the C-arm executes a pre-set rotational sweep with automatic image capture and on-system volumetric reconstruction. Key acquisition parameters, such as dose level, frame rate, rotation range, and contrast injection settings, are selected in advance.

At our institution, 3D-RA is acquired using a standardized protocol with fixed geometry and acquisition settings (122 images, LAO 103°, 30 fps, SID 120 cm). Despite consistent nominal parameters, the recorded dose metrics (DAP and Ka,r) exhibit minor variation between runs. This variation is attributable to patient-dependent factors, such as body habitus, positioning, and attenuation, as well as the behavior of the automatic exposure control, which adapts tube output, including tube current and pulse width, to maintain image quality while minimizing radiation dose.

Of the 132 procedures, 3D-RA was performed in 88 cases (66.6%). It was acquired once in 49 procedures, twice in 36 procedures, three times in three procedures, and four times in one procedure. For each 3D-RA acquisition, the mean DAP and Ka,r were 3.99 ± 0.70 Gy·cm^2^ and 11.1 ± 2.2 mGy, respectively.

Most 3D-RA acquisitions (93.2%; 82 out of 88) were performed using single-plane imaging.

Table 3 summarizes the utilization patterns of 3D-RA in the analyzed endovascular procedures. It presents the percentage distribution of cases in which 3D-RA was performed, the frequency of acquisitions per procedure (single versus multiple acquisitions), and the proportion of 3D-RA use in treatments conducted with a single X-ray tube/frontal projection compared to those performed with a dual-tube system that enables simultaneous frontal and lateral projection.

### 2.5. Computed Tomography Scan

Routine non-contrast head computed tomography (CT) was performed within 24 h of endovascular treatment to enable early identification of post-procedural complications. The primary purpose of this control imaging was to rule out acute intracranial hemorrhage, cerebral infarction, or clinically relevant cerebral edema related to the intervention. Additionally, the scan was used to verify the stability of the embolic material and to provide a baseline for interpreting any subsequent neurological deterioration.

The angiography suite functions as a hybrid operating room equipped for both endovascular and neurosurgical procedures, which enables intra-procedural CT imaging when clinically indicated. In this series, intra-procedural CT was utilized in 4 of 132 interventions to support ventricular drainage implantation.

The mean DAP per CT acquisition was 28.4 ± 0.4 Gy·cm^2^, and the mean Ka,r was 67.5 ± 1.3 mGy.

### 2.6. Intraoperative Somatosensory Evoked Potential Monitoring

Following the institution’s introduction of somatosensory evoked potential (SSEP) intraoperative monitoring in 2024, SSEP has been routinely applied during diagnostic and therapeutic evaluations for complex cerebrovascular disease and is generally required for interventional cases. In 2024, SSEP was used in 18 stent-assisted endovascular procedures; however, this sample size is insufficient for meaningful statistical analysis. Nevertheless, the 75th percentile (P75) dose metrics for procedures with SSEP were comparable to those in the overall angiography cohort (DAP: 20.23 vs. 19.89; Ka,r: 273 vs. 236). These preliminary findings indicate that implementing SSEP monitoring does not increase radiation dose during diagnostic procedures. Further studies are underway to validate this observation in a larger cohort.

### 2.7. Statistical Analysis

Consistent with International Commission on Radiological Protection (ICRP) Publication 135 [9], all available observations were retained for analysis. Due to substantial dispersion and large standard deviations (SDs) in dose metrics, the data were log-transformed (log10), yielding distributions closer to normal.

Statistical analysis was conducted in Python 3.12 using the pandas 2.3.3 and SciPy 1.16.2 libraries, with visualizations generated using matplotlib 3.10.3. Given the observational and non-randomized study design, the analytical approach was primarily descriptive. Continuous variables were summarized as mean ± SD with range, while skewed distributions were reported as median (P50) with interquartile range (P25-P75). Normality was assessed using the Shapiro–Wilk test. Between-group comparisons were performed using Student’s t-test for approximately normally distributed data, the Mann–Whitney U test for non-parametric independent samples, and Wilcoxon’s signed-rank test for paired data. Statistical significance was defined as *p* < 0.05 (95% confidence level).

## 3. Results

Table 4 provides descriptive statistics for the entire cohort of intracranial aneurysm stent procedures, with or without adjunctive coil embolization (132). Reported metrics include typical values (P50), local diagnostic reference levels (P75), and means and SDs for DAP, Ka,r, FT, and the number of DSA frames per procedure. The sample size of each treatment subgroup (including adjunctive coiling and stent number/placement categories) is provided in Table 2.

Analysis of DAP, Ka,r, FT, and the number of DSA frames revealed that all parameters were significantly higher in procedures performed with two X-ray tubes (*n* = 48) than in those performed with a single tube (*n* = 84).

Comparative analysis of DAP, Ka,r, FT, and the number of DSA frames between women (*n* = 105) and men (*n* = 27) showed higher DAP, Ka,r, and DSA frame counts in men, whereas FT did not differ significantly between sexes (Table 4). Given the small number of male patients, these comparisons should be interpreted with caution due to limited statistical power. The observed differences may be partly explained by differences in the distribution of single-plane imaging. Specifically, 15/27 men were treated with a single-plane configuration, while the majority of women (69/84) were also treated in this mode. This distribution could have contributed to lower dose metrics in women. Additionally, the groups differed substantially in anthropometric characteristics: mean body weight was 71.9 ± 14.9 kg in women and 85.0 ± 15.81 kg in men, while mean height was 1.64 ± 0.06 m in women and 1.75 ± 0.08 m in men, potentially influencing radiation exposure parameters.

A biplane imaging system (frontal and lateral C-arms) was used in 48 of 132 intracranial aneurysm embolization procedures (36.4%). Procedures performed exclusively in the frontal projection were associated with significantly lower radiation exposure than those performed with both frontal and lateral projections. As shown in Table 3, most 3D-RA acquisitions were conducted in single-plane mode (93.2%; 82 of 88; *p* < 0.05). In these cases, the operator relied on 3D reconstructions and roadmapping for spatial orientation and procedural guidance, which typically allowed the omission of an additional lateral projection. These findings suggest that the use of 3D-RA may reduce patient radiation exposure by decreasing the number of required projections.

These observations are consistent with prior reports by the authors on local DRLs for diagnostic cerebral angiography and for coil-only aneurysm embolization [10].

Overall, radiation appears to be strongly influenced by the number of projections required for reliable visualization of vascular pathology. The selection between single-plane and biplane imaging was further determined by clinical and anatomical factors, such as lesion location, vessel diameter, vessel course, and aneurysm morphology, which may account for higher dose levels in more complex cases. The data also suggest that increased aneurysm morphological complexity may be associated with higher X-ray exposure; however, this relationship requires confirmation in future studies.

To further characterize the study population, a dedicated subgroup of patients treated with a single stent and adjunctive coil embolization was identified. Table 4 includes a subgroup of 104 patients who received a single stent and adjunctive coils. The values of DAP, Ka,r, FT, and the number of DSA frames in this subgroup did not differ significantly from those observed in the overall patient cohort (*p* < 0.05, 95% confidence level).

## 4. Discussion

Establishing DRLs for endovascular treatment of intracranial aneurysms poses significant challenges, primarily due to limited procedural reproducibility and substantial heterogeneity in treatment techniques and vascular lesions. Variations in aneurysm size, location, and morphology, as well as overall procedural complexity, contribute to this heterogeneity. To enhance cohort homogeneity and enable a more meaningful assessment of radiation exposure, a subgroup was delineated comprising aneurysm treatments performed with stents alone or with stents and coils (stent-assisted coiling). This approach narrowed the analytical scope and facilitated a more focused interpretation of DRL results for this subgroup.

Our previous studies established local DRLs for diagnostic cerebral angiography [11] and aneurysm embolization using coils only [10]. In both analyses, a low-dose strategy was implemented, resulting in patient radiation exposure metrics that were comparable to the lowest values reported in the literature over the past decade (Table 5).

Importantly, most publications addressing DRLs in endovascular treatment of cerebral aneurysms do not stratify results by technique (e.g., stent, stent-assisted coiling, coils alone), which substantially limits direct comparisons [14,15,16,17,18,19,20,21,22,23,24,25,26,27]. A summary of these studies is provided in Table 5. In this context, the values obtained in our study are substantially lower: the P75 for DAP was 19.89 Gy·cm^2^, and the P75 for Ka,r was 332 mGy. By comparison, the P75 values for DAP reported in the studies summarized range from 123 to 272.8 Gy·cm^2^, while the corresponding P75 values for Ka,r range from 1171 to 4240 mGy. Comparability is further constrained by differences in case mix, angiography system generation and configuration, and acquisition settings as well as reporting conventions and the inclusion/exclusion of adjunct imaging (e.g., intraprocedural CT).

DRLs for flow-diverter procedures have also been reported [12,22,28]; however, studies that clearly separate a “stent plus coils” subgroup remain scarce. Rai et al. [12] reported such a subgroup in a study primarily focused on woven endovascular bridge (WEB) treatment. Since WEB eligibility selects cases based on aneurysm anatomy and morphology, direct comparison with the present cohort is limited. In Rai et al. [12], the median Ka,r for the stent-assisted embolization subgroup was 3442 mGy, and the FT was 6 min (*n* = 29). Ihn et al. [13] evaluated a partially comparable population but included both stent-assisted coiling and balloon-assisted coiling, further limiting comparability; in that study, the median Ka,r was 2104 mGy and the FT was 40.9 min. Notably, FT does not directly correspond to Ka,r, as Ka,r is also influenced by the number and type of acquisitions (e.g., DSA, 3D-RA), system geometry, and collimation. These parameters may vary substantially across centers and studies, even when FT is similar.

Despite these limitations, the published evidence may serve as an approximate benchmark for overall exposure levels in aneurysm treatment. Against this background, our results, encompassing more complex cases requiring stent-assisted coiling, appear lower than many values reported in Table 5, acknowledging the limited cross-study comparability. Moreover, our previously established DRLs based on 245 coil-only embolization procedures were comparable to the exposure levels observed in the present stent-assisted coiling analysis (Table 5). We observed these findings in association with the consistent implementation of the low-dose technique within a standardized workflow on a modern biplane system.

According to Pearl et al. [7], the total ionizing radiation dose during a single procedure is determined by multiple interacting factors, including patient characteristics (e.g., body habitus), vascular anatomy, clinical indications, technical parameters, and operator-dependent procedural efficiency. Key technical determinants include the number of DSA acquisitions per procedure, DSA frame rate, fluoroscopy frame rate, detector technology (image intensifier or flat-panel detector), collimation strategy, and system geometry, particularly the source-to-image distance (SID).

In our cohort, imaging mode was associated with procedural dose: procedures performed with single-plane acquisition when feasible showed lower DAP/Ka,r than those requiring biplane imaging (Table 4). This is expected from a physical and workflow perspective, as biplane operation activates an additional X-ray tube/detector chain and may increase the cumulative acquisition burden (e.g., total frames and runs), thereby increasing patient exposure. Importantly, biplane imaging remains indispensable in complex anatomy and device navigation; therefore, our findings support a pragmatic strategy of preferential single-plane use when clinically appropriate, with escalation to biplane imaging dictated by procedural complexity rather than as a default setting.

Operator experience is also essential, including avoiding fluoroscopy during phases without active catheter/guidewire manipulation and deliberately applying exposure-reduction strategies [29]. In addition, physicians performing these procedures should be familiar with the angiography system specifications and default DSA acquisition settings [30]. Kahn et al. [31] demonstrated that modifying factory-default system configurations can substantially reduce exposure. The literature indicates that lowering fluoroscopy frame rate from 15 to 7.5 fps is associated with a significant reduction in radiation dose for both patients and staff [7,30,32]. Furthermore, flat-panel detector systems have enabled exposure reductions to approximately 30% compared with older image intensifier systems [33]. From an organizational perspective, routine intraprocedural head CT was not performed; it was used only when an external ventricular drain was implanted. Additional reductions were achieved by limiting the number of acquisitions and the number of catheterized vessels based on prior imaging (CT angiography [CTA], magnetic resonance angiography [MRA], DSA), thereby avoiding repeated assessment of the aortic arch and carotid bifurcations and focusing the procedure on vessels supplying a known lesion, particularly during follow-up examinations.

Three-dimensional rotational angiography (3D-RA) is typically more dose intensive than a short fluoroscopy run; however, selective 3D-RA use may improve spatial understanding of aneurysm geometry and facilitate working-projection selection, potentially reducing the need for repeated DSA acquisitions and prolonged fluoroscopy. This potential “downstream” dose-saving effect may be particularly relevant in workflows that favor single-plane imaging, where accurate projection planning can partially compensate for the lack of simultaneous orthogonal views. Consequently, the relationship between 3D-RA frequency and total dose is not necessarily linear and likely depends on case complexity and the number of subsequent acquisitions avoided.

Operating at reduced frame rates primarily affects temporal resolution, resulting in less “smooth” image sequences rather than a deterioration of image quality per se. Familiarity with low frame-rate imaging increases operator tolerance to reduced sequence smoothness and improves the spatial perception required for precise visualization of vascular pathology. In practice, stepwise implementation, such as performing early procedural phases at lower frame rates until the target lesion is reached, facilitates feasibility assessment and allows comparison of dose-report metrics from procedures partly performed at low frame rates with those from earlier routine procedures.

In future prospective studies, a pragmatic stepwise implementation (e.g., protocol roll-out over time or matched historical comparisons) may help quantify dose effects while avoiding non-beneficial dose escalation solely for research purposes.

Dose reduction is beneficial provided it does not compromise procedural diligence and precision. Lower patient exposure also translates into reduced occupational exposure for the operator, who typically receives the highest scattered radiation dose within the interventional team.

## 5. Limitations

This study was designed as a single-center, retrospective dose audit with DRL analysis, which inherently limits generalizability and causal inference. All procedures were performed by a single highly experienced neurointerventionalist with long-standing familiarity with low-frame-rate imaging; therefore, reproducibility in less specialized settings and among less experienced operators remains uncertain and the reported DRLs should be interpreted as local benchmarks requiring external validation.

No contemporaneous control group using standard frame-rate protocols was available, which precludes attributing the observed low dose levels specifically to the low-dose strategy or to frame-rate reduction alone. The low-dose configuration represented routine clinical practice on the evaluated system throughout the study period, and conventional frame-rate settings were not used. A prospective protocol-comparison study involving dose escalation in a subgroup solely for research purposes was not undertaken, as it would have exposed patients to additional radiation without direct clinical benefit. Future prospective, pragmatic comparisons (e.g., step-wedge implementation, matched historical controls, or multicenter protocol comparisons) may help disentangle the contributions of individual dose-reduction components. Moreover, several subgroup analyses included small sample sizes (e.g., the male subgroup), potentially limiting statistical power; these findings should therefore be interpreted as exploratory.

All procedures were performed on a single biplane angiography platform employing vendor-specific dose-reduction and dose-optimization features, limiting transferability to other systems and configurations. Multicenter, multi-operator validation is required before these DRLs can be considered as broader reference benchmarks.

Radiation exposure was assessed only for the endovascular procedure itself. Follow-up CT examinations performed during hospitalization were not included and should be considered when evaluating cumulative patient exposure.

No formal, blinded, multi-reader image-quality assessment was performed to determine how reduced frame rates may affect operator-perceived image quality and angiographic interpretation. Accordingly, statements regarding preserved image adequacy reflect routine clinical acceptability and operator experience rather than objective validation. In addition, the retrospective dataset lacked structured information on aneurysm morphology, lesion size, the number of coils used, or neck-to-dome geometry, and did not provide objective procedural complexity metrics or cranial anthropometric parameters. Consequently, multivariable adjustment and quantitative assessment of potential confounding variables were limited. We also did not evaluate a learning-curve effect or temporal trends in dose over the study period; such analyses would require a dedicated design with case-mix control.

This dose audit did not include structured clinical outcome measures (e.g., technical success, complication rates, occlusion grading, or functional outcomes); therefore, the findings should be interpreted primarily as feasibility and dose-performance results rather than evidence of unchanged efficacy or safety.

Finally, at our center, stent-only treatment or stent-assisted coiling is typically reserved for aneurysms with unfavorable anatomy, including wide-neck lesions; therefore, case selection should be considered when comparing our results with more heterogeneous endovascular series.

## 6. Conclusions

This single-center retrospective dose audit of endovascular treatment of intracranial aneurysms using stent-only techniques or stent-assisted coiling on a modern biplane angiography system with a dedicated low-dose configuration established local DRLs for DAP and Ka,r that fall within the lower range of dose metrics reported in the literature. However, comparisons with published data are descriptive and should be interpreted with caution due to substantial methodological heterogeneity across studies and differences in equipment, protocols, operator practice, and case mix.

The low exposure levels observed were observed in association with the combined use of very low fluoroscopy and DSA frame rates, advanced dose-reduction technology, and extensive operator experience in low-dose imaging. These findings demonstrate the feasibility that, within a consistent, standardized workflow, low procedural doses can be achieved and local DRLs can be defined in appropriately selected cases treated with stents (with or without coils). Nevertheless, this retrospective audit was not designed to evaluate clinical safety endpoints, or procedural effectiveness, assess formally evaluate a learning curve, nor to isolate the independent contribution of frame-rate reduction from other determinants, including equipment configuration, operator practice, and case selection. In addition, no formal multi-reader image-quality assessment was performed, and no contemporaneous standard-frame-rate control group was available; therefore, causal attribution and statements beyond dose-performance and feasibility should be avoided. Further multicenter, multi-operator studies are required to confirm these observations and to support broader implementation of ambitious local DRLs.

## Figures and Tables

**Figure 2:**
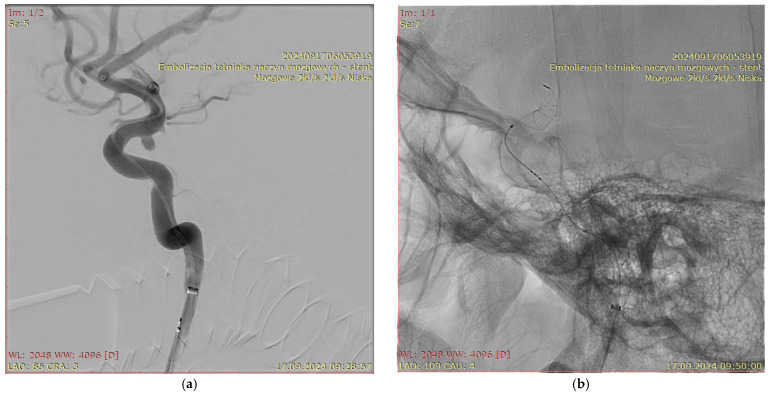
An angiographic image of the intracranial aneurysm found in Patient X (**a**) and a fluoroscopic X-ray image acquired during stent deployment (**b**) “mózgowe 2 kl/s, 2 kl/s niska” (“Cerebral 2 fps, 2 fps Low”), “embolizacja tetniaka naczyn mozgowych- stent” (“Endovascular embolization of a cerebral aneurysm- stent”).

**Figure 3:**
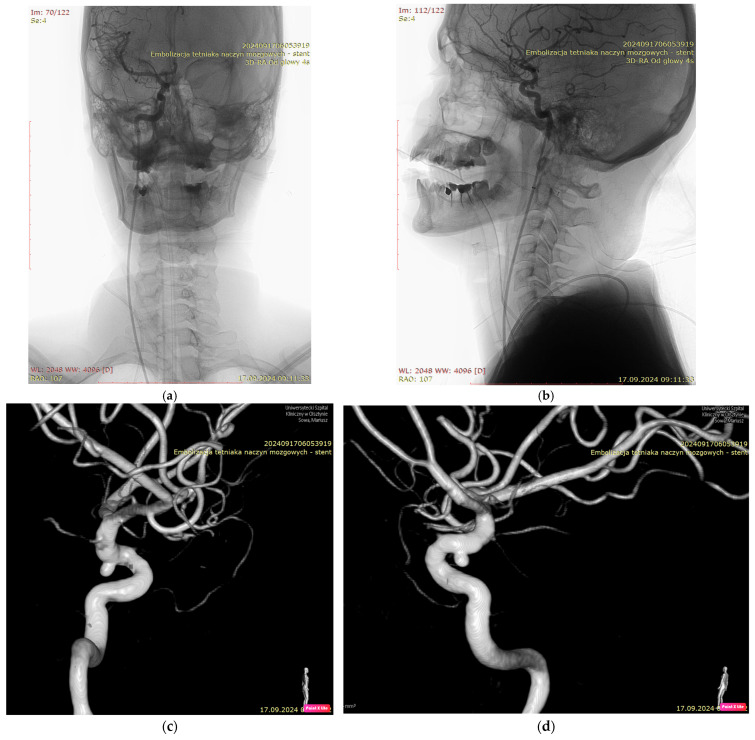
Three-dimensional rotational angiography (3D-RA) of the head of Patient X. An anterior 3D-RA view (**a**), a lateral 3D-RA view (**b**), a lateral reconstruction (**c**), and an oblique reconstruction (**d**) “Uniwersytecki Szpital Kliniczny w Olsztynie” (University of Warmia and Mazury in Olsztyn, Mariusz Sowa- operator’s last name”) “3D-RA Od głowy” (“3D-RA head”), “embolizacja tetniaka naczyn mozgowych- stent” (“Endovascular embolization of a cerebral aneurysm- stent”).

**Figure 4:**
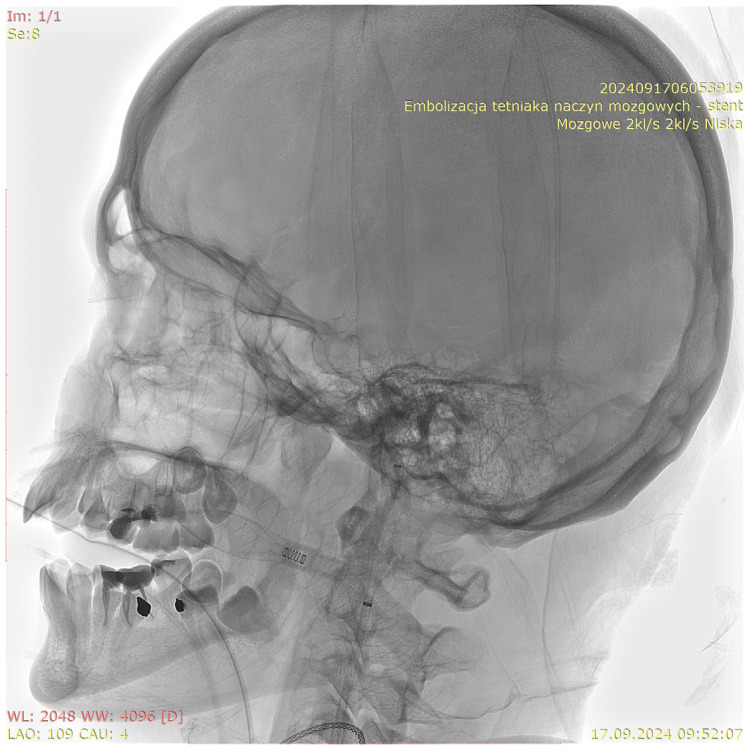
A three-dimensional rotational angiography (3D-RA) reconstruction demonstrating the fully deployed stent within the target vessel in Patient X “mózgowe 2 kl/s, 2 kl/s niska” (“Cerebral 2 fps, 2 fps Low”), “embolizacja tetniaka naczyn mozgowych- stent” (“Endovascular embolization of a cerebral aneurysm- stent”).

**Figure 6:**
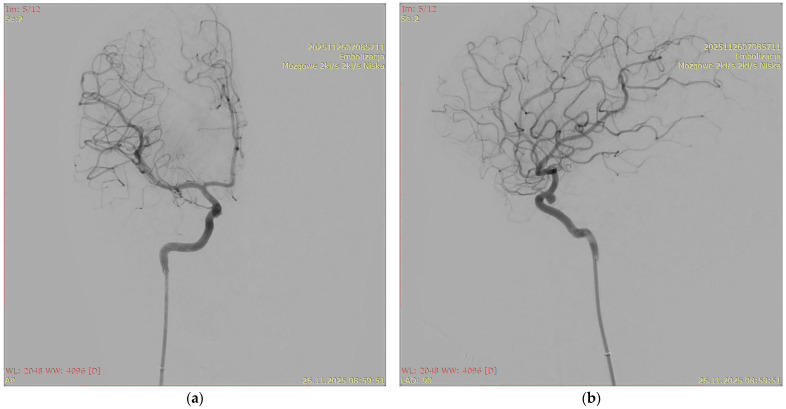
Anterior (**a**) and posterior (**b**) angiographic images of the intracranial aneurysm in Patient Y before treatment “mózgowe 2 kl/s, 2 kl/s niska” (“Cerebral 2 fps, 2 fps Low”), “embolizacja” (“Endovascular embolization”).

**Figure 7:**
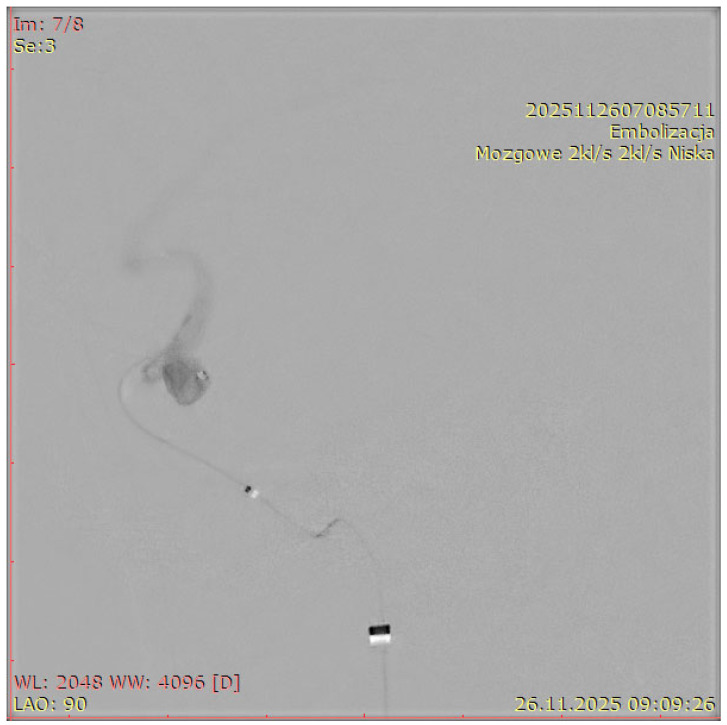
Aneurysmography of a cervical artery aneurysm in Patient Y “mózgowe 2 kl/s, 2 kl/s niska” (“Cerebral 2 fps, 2 fps Low”), “embolizacja” (“Endovascular embolization”).

**Figure 8:**
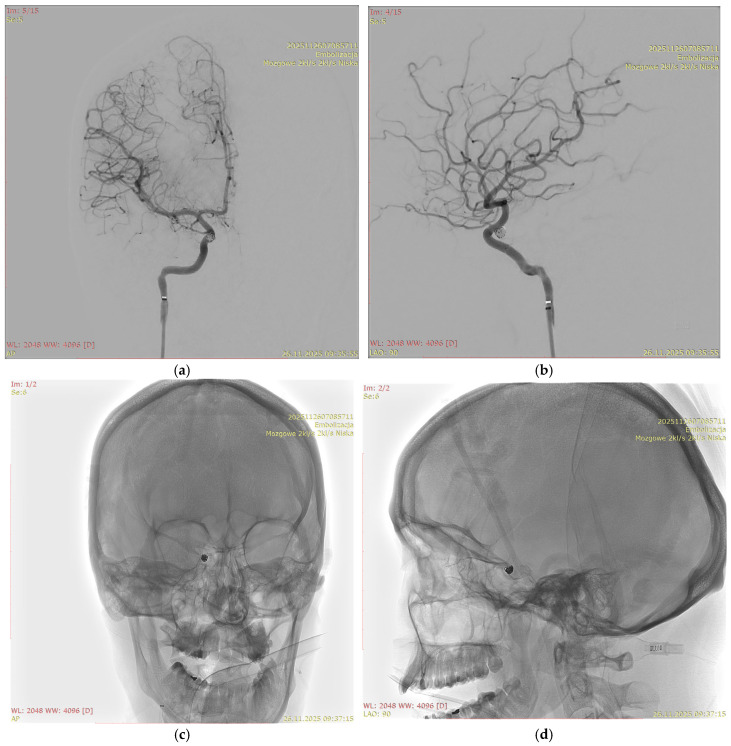
Anterior (**a**) and posterior (**b**) angiographic images of the intracranial aneurysm in Patient Y after treatment. Anterior (**c**) and lateral (**d**) view three-dimensional rotational angiography (3D-RA) of the head “mózgowe 2 kl/s, 2 kl/s niska” (“Cerebral 2 fps, 2 fps Low”), “embolizacja” (“Endovascular embolization”).

**Table 1:** Number of aneurysm treatment procedures by sex, age, and height.

Variable	Female	Male	Total
Mean age [years] ± SD	53.9 ± 11.3	54.1 ± 15.48	54.0 ± 12.1
Mean height [m] ± SD	1.64 ± 0.06	1.75 ± 0.08	1.66 ± 0.08
Mean body weight [kg] ± SD	71.9 ± 14.9	85 ± 15.81	74.3 ± 15.8
Number of procedures	105	27	132

**Table 2:** Treatment characteristics, including the number of stents used, treatment technique (telescopic placement or separate implantation sites), number of aneurysms treated, use of adjunctive coil embolization, and the number of patients in each category.

Number of Stents Used	Treatment Characteristics	Number of Aneurysms Treated	Adjunctive Coil Embolization	Number of Patients in Each Category
2	Separate implantation sites	2	Yes	1
1	-	2	No	1
2	Telescopic placement	1	No	1
2	Telescopic placement	2	Yes	1
2	Telescopic placement	3	Yes	1
2	Separate implantation sites	2	Yes	23
1	-	2	Yes	104

**Table 3:** Percentage distribution of 3D-rotational angiography (3D-RA) utilization in procedures, including the number of 3D-RA acquisitions performed per treatment, stratified by the number of X-ray tubes/projections (single vs. dual).

Three-Dimensional Rotational Angiographies Per Procedure	X-Ray Tubes/Projections		
	Frontal Only	Frontal and Lateral	All
4	1/84	0	1
3	3/84	0	3
2	33/84	3/48	36
1	45/84	4/48	49
0	2/84	41/48	43
% all (132)	63.6% (84)	36.4% (48)	132
% all with 3D-RA (88)	93. 2% (82)	6.8% (6)	88

**Table 4:** Results for all 132 aneurysm treatments, subdivided by sex (female and male), X-ray configuration (frontal lamp only versus frontal and lateral lamp), treatment technique (single stent with coils), and use of intraoperative somatosensory evoked potential (SSEP) monitoring.

Variable	Nr	Dose-Area Product			*p*	Reference Air Kerma			*p*	Number of Images			*p*	Fluoroscopy Time (seconds)			*p*
		P50	P75	Mean ± SD		P50	P75	Mean ± SD		P50	P75	Mean ± SD		P50	P75	Mean ± SD	
All cohort	132	13.71	19.89	16.79 ± 10.43	*p* = 0.67	219.5	332	273.27 ± 197.28	*p* = 0.63	277.5	354.0	300.3 ± 143.94	*p* = 0.60	1236.0	1532.5	1389.17 ± 607.73	*p* = 0.46
One Stent and Coils	104	14.00	19.80	16.90 ± 10.06		223	351	280.44 ± 194.42		279	354	303.94 ± 139.51		1267	1557	1436.00 ± 613.73	
Female	105	12.25	16.73	14.48 ± 7.31	*p* < 0.05	188	302	234.48 ± 136.45	*p* < 0.05	258	339	278.92 ± 116.81	*p* < 0.05	1197	1465	1321.58 ± 502.80	*p* = 0.57
Male	27	21.12	32.16	25.79± 15.14		335	517.5	424.11 ± 303.16		348	448.5	383.44 ± 201.98		1337	1754	1652 ± 871.45	
Frontal Lamp Only	84	12.19	16.73	14.50 ±6.92	*p* < 0.05	159	229	197.50 ± 110.28	*p* < 0.05	297	350	299.07 ± 84.52	*p* < 0.05	1210	1445	1289.14 ± 426.84	*p* < 0.05
Frontal and Lateral Lamp	48	17.02	26.09	20.93 ± 13.97		351	472	410.30 ± 242.55		236	368.5	302.53 ± 214.35		1309	1901.5	1570.06 ± 816. 80	
Procedures with SSEP	18	14.35	20.23	18.27 ± 11.12	-	224.5	236.5	236.68 ± 184.48	-	272.5	361.5	333.83 ± 184.48	-	1218.5	1404.5	1208.89 ± 286.72	-

**Table 5:** Diagnostic reference level studies for cerebral embolization.

Authors	Study Period	Participants and Treatments	Study Type	Dose-Area Product (Gy·cm^2^)		Reference Air Kerma (mGy)		Fluoroscopy Time		Number of Frames	
				P50	P75	P50	P75	P50	P75	P50	P75
This study	June 2018–December 2024	132	Local	13.71	19.85	219.5	332	20 min, 36 s	25 min, 32 s	277.5	354.0
Rai et al. (2021) [12]		46 WEB 41 SAC *	local			2392 3442		34 min			
Ihn et al. 2021 [13]	December 2020–June 2021	327	local	130.6	199.6	2104	3458.7	40.9 min	57.3 min		
Sowa et al. (2026) [10]	June 2018–December 2024	245 coil only	Local	13.8	22.4	196.0	268.0	13 min, 25 s	18 min, 58 s	208	285
Sowa et al. (2025) [11]	June 2018–December 2024	213 angiography	Local	13.20	23.57	92	153	6 min 20 s	9 min 9 s		
Kanda et al. (2021) [14]	-	-	National		210		3100				
Hassan et al. (2017) [15]	2015	71	Local	78.7		1040		25.7 min		300	
Ihn et al. 2016 [16]	October 2016– December 2016	371	Multicenter	179.0	271.0	2804.0	4471.3	44.5 min	64.7 min	412.5	567.3
Tristram et al. (2022) [17]	June 2015–April 2018	129	Local	132.8	186.8	1397	1906	51.4 min	70 min		
Acton et al. (2018) [18]	November 2014–September 2016	109	Local	100	123						
Aly et al. (2024) [19]	January 2019–March 2020	local	Local	85	124	801	1171	19.5 min	35 min	717	1374
Opitz et al. (2023) [20]	2010–2021	583	Local	157	217	-	-	32.7 min	58 min		
Schegerer et al. (2019) [21]	2016–2018		Local	121	192			34 min	54 min		
Forbrig et al. (2020) [22]	January 2015–May 2019	26	Local	94				49 min			
Etard et al. (2017) [23]	2016	427	National	130	190	1718	2770	37.2 min	58 min		
Isoardi et al. (2019) [24]	January 2015–January 2019	489		164.5		2197		32.1 min			
Chun et al. (2014) [25]		111	Local	-	272.8				61.1 min		276
Rizk et al. (2021) [26]	March 2016–December 2019	39	Local	91		1113		15 min		288	
Choi et al. (2019) [27]	January 2012–June 2014		Local		206.2		4240		60 min		334

* stent assisted coiling.

## Data Availability

Department of Neurosurgery, CM UWM, Aleja Warszawska 30, 10-082 Olsztyn, Poland.

## Abbreviations

The following abbreviations are used in this manuscript:

DAP	The dose-area product, calculated as the cumulative air kerma (CAK) multiplied by the exposed area, and measured in Gy·cm^2^.
K_a,r_	Also known as reference point air kerma and formerly called cumulative dose or cumulative air kerma (CAK), it is the dose to air at the interventional reference point, which is 15 cm from the isocenter toward the x-ray tube. Ka,r is a cumulative approximation of the total radiation dose to the skin, summed over the entire procedure, and is measured in Gy·cm^2^.
DSA	Digital subtraction angiography.
FT	Fluoroscopy time.
DRL	Diagnostic reference level.
RTG	X-ray radiation, ionizing radiation, or Roentgen radiation.
SD	Standard deviation.

## References

[1] Ribeiro A., Husson O., Drey N., Murray I., May K., Thurston J., Oyen W. (2020). Ionising radiation exposure from medical imaging—A review of Patient’s (un) awareness. Radiography.

[2] Zanzonico P.B. (2016). The Neglected Side of the Coin: Quantitative Benefit-Risk Analyses in Medical Imaging. Health Phys..

[3] Frane N., Bitterman A. (2025). Radiation Safety and Protection. StatPearls.

[4] Greenberg E., Katz J.M., Janardhan V., Riina H., Gobin Y.P. (2007). Treatment of a giant vertebrobasilar artery aneurysm using stent grafts. Case report. J. Neurosurg..

[5] Karavas E., Ece B., Aydın S., Kocak M., Cosgun Z., Bostanci I.E., Kantarci M. (2022). Are we aware of radiation: A study about necessity of diagnostic X-ray exposure. World J. Methodol..

[6] Gailloud P. (2015). A large display is a powerful tool to reduce radiation exposure during single-plane fluoroscopically guided procedures. AJR Am. J. Roentgenol..

[7] Pearl M.S., Torok C., Wang J., Wyse E., Mahesh M., Gailloud P. (2015). Practical techniques for reducing radiation exposure during cerebral angiography procedures. J. NeuroInterventional Surg..

[8] Sowa M. (2022). Leczenie Wewnątrznaczyniowe Małych Tętniaków Naczyń Mózgowych.

[9] Vañó E., Miller D.L., Martin C.J., Rehani M.M., Kang K., Rosenstein M., Ortiz-López P., Mattsson S., Padovani R., Rogers A. (2017). ICRP Publication 135: Diagnostic Reference Levels in Medical Imaging. Ann. ICRP.

[10] Sowa M., Sowa J., Węglarz K., Budzanowski M. (2026). Local Diagnostic Reference Levels for Intracranial Aneurysm Coil-Only Embolization Using a Low-Dose Technique. Biomedicines.

[11] Sowa M., Sowa J., Węglarz K., Budzanowski M. (2025). Significant dose reduction in diagnostic cerebral angiography using the ALARA principle and state-of-the-art medical equipment. J. Radiol. Prot..

[12] Rai A.T., Turner R.C., Brotman R.G., Boo S. (2021). Comparison of operating room variables, radiation exposure and implant costs for WEB versus stent assisted coiling for treatment of wide neck bifurcation aneurysms. Interv. Neuroradiol..

[13] Ihn Y.K., Kim B.S., Jeong H.W., Suh S.H., Won Y.D., Lee Y.J., Kim D.J., Jeon P., Ryu C.W., Suh S.I. (2021). Monitoring Radiation Doses during Diagnostic and Therapeutic Neurointerventional Procedures: Multicenter Study for Establishment of Reference Levels. Neurointervention.

[14] Kanda R., Akahane M., Koba Y., Chang W., Akahane K., Okuda Y., Hosono M. (2021). Developing diagnostic reference levels in Japan. Jpn. J. Radiol..

[15] Hassan A.E., Amelot S. (2017). Radiation Exposure during Neurointerventional Procedures in Modern Biplane Angiographic Systems: A Single-Site Experience. Interv. Neurol..

[16] Ihn Y.K., Kim B.S., Byun J.S., Suh S.H., Won Y.D., Lee D.H., Kim B.M., Kim Y.S., Jeon P., Ryu C.W. (2016). Patient Radiation Exposure During Diagnostic and Therapeutic Procedures for Intracranial Aneurysms: A Multicenter Study. Neurointervention.

[17] Tristram J., Steuwe A., Kröpil F., Thomas C., Rubbert C., Antoch G., Boos J. (2022). Typical doses and typical values for fluoroscopic diagnostic and interventional procedures. J. Radiol. Prot..

[18] Acton H., James K., Kavanagh R.G., O’Tuathaigh C., Moloney D., Wyse G., Fanning N., Maher M., O’Connor O.J. (2018). Monitoring neurointerventional radiation doses using dose-tracking software: Implications for the establishment of local diagnostic reference levels. Eur. Radiol..

[19] Aly A., Tsapaki V., Ahmed A.Z., Own A., Patro S., Al Naemi H., Kharita M.H. (2024). Clinical diagnostic reference levels in neuroradiology based on clinical indication. Radiat. Prot. Dosim..

[20] Opitz M., Zenk C., Zensen S., Bos D., Li Y., Styczen H., Oppong M.D., Jabbarli R., Hagenacker T., Forsting M. (2023). Radiation dose and fluoroscopy time of aneurysm coiling in patients with unruptured and ruptured intracranial aneurysms as a function of aneurysm size, location, and patient age. Neuroradiology.

[21] Schegerer A., Loose R., Heuser L.J., Brix G. (2019). Diagnostic Reference Levels for Diagnostic and Interventional X-Ray Procedures in Germany: Update and Handling. Rofo.

[22] Forbrig R., Ozpeynirci Y., Grasser M., Dorn F., Liebig T., Trumm C.G. (2020). Radiation dose and fluoroscopy time of modern endovascular treatment techniques in patients with saccular unruptured intracranial aneurysms. Eur. Radiol..

[23] Etard C., Bigand E., Salvat C., Vidal V., Beregi J.P., Hornbeck A., Greffier J. (2017). Patient dose in interventional radiology: A multicentre study of the most frequent procedures in France. Eur. Radiol..

[24] Isoardi P., D’Ercole L., Cavallari M., Gianusso L., Pini S., Giordano C., Angelini L., Colombo P.E., Canne S.D., Vecchio A.D. (2019). Patient dose in angiographic interventional procedures: A multicentre study in Italy. Phys. Med..

[25] Chun C.W., Kim B.-S., Lee C.H., Ihn Y.K., Shin Y.-S. (2014). Patient Radiation Dose in Diagnostic and Interventional Procedures for Intracranial Aneurysms: Experience at a Single Center. Korean J. Radiol..

[26] Rizk C., Abi Chedid G., Salem C., Farah J. (2021). Investigating the parameters that affect the radiation exposure and establishing typical values based on procedure complexity for cerebral angiography and brain aneurysm embolization. Neuroradiology.

[27] Choi J., Kim B., Choi Y., Shin N.Y., Jang J., Choi H.S., Jung S.L., Ahn K.J. (2019). Image Quality of Low-Dose Cerebral Angiography and Effectiveness of Clinical Implementation on Diagnostic and Neurointerventional Procedures for Intracranial Aneurysms. AJNR Am. J. Neuroradiol..

[28] Gärtner F., Klintz T., Peters S., Bueno Neves F., Mostafa K., Mahnke J., Hensler J., Flüh C., Larsen N., Jansen O. (2025). Intra-cranial aneurysm treatment with contour or WEB—A single center comparison of intervention times and learning curves. Interv. Neuroradiol..

[29] Levitt M.R., Osbun J.W., Ghodke B.V., Kim L.J. (2013). Radiation dose reduction in neuroendovascular procedures. World Neurosurg..

[30] Wali A.R., Pathuri S., Brandel M.G., Sindewald R.W., Hirshman B.R., Bravo J.A., Steinberg J.A., Olson S.E., Pannell J.S., Khalessi A. (2024). Reducing frame rate and pulse rate for routine diagnostic cerebral angiography: ALARA principles in practice. J. Cerebrovasc. Endovasc. Neurosurg..

[31] Kahn E.N., Gemmete J.J., Chaudhary N., Thompson B.G., Chen K., Christodoulou E.G., Pandey A.S. (2016). Radiation dose reduction during neurointerventional procedures by modification of default settings on biplane angiography equipment. J. Neurointerv Surg..

[32] Schneider T., Wyse E., Pearl M.S. (2017). Analysis of radiation doses incurred during diagnostic cerebral angiography after the implementation of dose reduction strategies. J. Neurointerv. Surg..

[33] Axelsson B. (2007). Optimisation in fluoroscopy. Biomed. Imaging Interv. J..

